# Genomic and proteomic evidences unravel the UV-resistome of the poly-extremophile *Acinetobacter* sp. Ver3

**DOI:** 10.3389/fmicb.2015.00328

**Published:** 2015-04-22

**Authors:** Daniel Kurth, Carolina Belfiore, Marta F. Gorriti, Néstor Cortez, María E. Farias, Virginia H. Albarracín

**Affiliations:** ^1^Laboratorio de Investigaciones Microbiologicas Lagunas Andinas, Centro Científico Tecnológico, Planta Piloto de Procesos Industriales Microbiológicos – Consejo Nacional de Investigaciones Científicas y Técnicas, San Miguel de TucumánArgentina; ^2^Centro Científico Tecnológico, IBR – CONICET, Universidad Nacional de RosarioRosario, Argentina; ^3^Facultad de Ciencias Naturales e Instituto Miguel Lillo, Universidad Nacional de Tucumán, San Miguel de TucumánArgentina

**Keywords:** *Acinetobacter*, genome, extremophiles, high-altitude Andean lakes, UV-resistance

## Abstract

Ultraviolet radiation can damage biomolecules, with detrimental or even lethal effects for life. Even though lower wavelengths are filtered by the ozone layer, a significant amount of harmful UV-B and UV-A radiation reach Earth’s surface, particularly in high altitude environments. high-altitude Andean lakes (HAALs) are a group of disperse shallow lakes and salterns, located at the Dry Central Andes region in South America at altitudes above 3,000 m. As it is considered one of the highest UV-exposed environments, HAAL microbes constitute model systems to study UV-resistance mechanisms in environmental bacteria at various complexity levels. Herein, we present the genome sequence of *Acinetobacter* sp. Ver3, a gammaproteobacterium isolated from Lake Verde (4,400 m), together with further experimental evidence supporting the phenomenological observations regarding this bacterium ability to cope with increased UV-induced DNA damage. Comparison with the genomes of other *Acinetobacter* strains highlighted a number of unique genes, such as a novel cryptochrome. Proteomic profiling of UV-exposed cells identified up-regulated proteins such as a specific cytoplasmic catalase, a putative regulator, and proteins associated to amino acid and protein synthesis. Down-regulated proteins were related to several energy-generating pathways such as glycolysis, beta-oxidation of fatty acids, and electronic respiratory chain. To the best of our knowledge, this is the first report on a genome from a polyextremophilic *Acinetobacter* strain. From the genomic and proteomic data, an “UV-resistome” was defined, encompassing the genes that would support the outstanding UV-resistance of this strain.

## Introduction

Ultraviolet radiation is one of the most limiting abiotic factors, causing diverse detrimental effects on living organisms depending on the radiation wavelength. There are three major subtypes of UV rays, namely, UV-A (320–400 nm), UV-B (280–320 nm), and UV-C (100–280 nm). UV-A accounts for about 95% of the total UV energy that reaches the Earth’s surface, the remaining 5% being UV-B; UV-C gets totally absorbed by stratospheric gasses, mainly oxygen and ozone, and thus fails to reach the troposphere (Agogué et al., 2005; Hernández et al., 2007). Regarding the biological effects of UV light, UV-C is the most detrimental to living cells, because its short wavelengths, especially 256 nm, are absorbed directly by DNA, causing formation of cyclobutane dimers and single-stranded breaks in the sugar-phosphate backbone. UV-B not only generates directly DNA photoproducts, but also produces reactive oxygen species (ROS), such as hydrogen peroxide (H_2_O_2_), superoxide and hydroxyl radicals that can induce single- and double- strand breaks, apurinic sites and base damage (Demple and Harrison, 1994). UV-A is the least energetic of the three types of UV radiation and acts indirectly by producing photooxidizing compounds and ROS that damage DNA, proteins, and lipids (Wilson et al., 2004). The low redox potential of guanine makes this base particularly vulnerable and leads to the generation of various oxidized guanine products (Neeley and Essigmann, 2006; David et al., 2007) such as 8-oxo-7,8-dihydroguanine (8-oxoG). This lesion has strong promutagenic properties (Demple and Harrison, 1994).

Many DNA modifications are deleterious for cells, promoting organisms to develop reparation mechanisms. Repair pathways might have evolved to protect cells against the effects of endogenous damage (Dalhus et al., 2009), but also toward injury inflicted by exogenous agents such as UV. Besides general pathways that address numerous types of DNA damage, such as the base excision repair (BER) system, there are also specific enzymes directed to one type of lesion, for example photolyases (Dalhus et al., 2009).

On environmental settings, UV irradiation is higher at high altitudes and the related cell damages are much more pronounced. On these regards, the effect of solar UV incidence was studied on plankton from alpine lakes (Williamson, 1995; Halac et al., 1997; Winter et al., 2001; Häder et al., 2007), on microbial diversity in the Himalayas (Liu et al., 2006; Jiang et al., 2007), and on extremophiles thriving at high-altitude Andean lakes (HAALs). These are a group of disperse shallow lakes and salterns, located at the Dry Central Andes region in South America at altitudes above 3,000 m, and considered one of the highest UV-exposed environments on Earth (Albarracín et al., 2011). Solar UV-B flux reaches 1.02 kJ m^-2^ per hour in some lakes compared with 0.006–0.024 kJ m^-2^ per hour at the sea level. The low impact of solar radiation on bacterioplankton diversity was shown for the hypersaline Andean lake Vilama (4,650 m asl), suggesting efficient adaptation mechanisms to high solar irradiance (Farías et al., 2009). Similar results were obtained regarding the microbial communities biodiversity of most lakes at the HAAL (Zenoff et al., 2006; Dib et al., 2009; Farías et al., 2009; Ordoñez et al., 2009). UV was also shown to modulate the metabolic activity and the dominant cyanobacteria distribution (*Microcoleus*) in the modern stromatolites from Lake Socompa (Farías et al., 2013).

In accordance to this highly extreme solar scenario, HAAL-isolated strains displayed an intrinsic and high UV-resistance. A set of almost one hundred UV-resistant strains were isolated and identified (Fernández Zenoff et al., 2006; Zenoff et al., 2006; Dib et al., 2008; Flores et al., 2009; Ordoñez et al., 2009; Bequer Urbano et al., 2013) in which *Acinetobacter* strains were the most abundant. Experimental evidence suggest a particularly high antioxidant activity (Di Capua et al., 2011) as well as an efficient DNA damage photorepairing ability (Albarracín et al., 2012), particularly in *Acinetobacter* sp. Ver3 strain. This polyextremophile, isolated from Lake Verde (4,400 m asl), was able to grow under high UV-B irradiation doses, in salt concentrations up to 10% (Flores et al., 2009; Ordoñez et al., 2009; Albarracín et al., 2011), and with arsenite up to 10 mM (unpublished data). For these reasons, Ver3 was chosen as model organism for performing comprehensive studies on the complex genetic and metabolic system shared by UV-resistant HAAL microbial communities, herein named as UV-resistome. Current model radiation resistant extremophiles are *Deinococcus* spp. (Makarova et al., 2001), *Halobacterium* spp. (Baliga et al., 2004; Crowley et al., 2006), and *Bacillus* spp. (Gioia et al., 2007). More recently, *Acinetobacter radioresistans* and other species have been isolated from spacecraft assembly rooms (McCoy et al., 2012; Derecho et al., 2014). All these strains were studied for their resistance to high-energy radiation, mainly gamma rays or UV-C; however, the mechanisms described may not be applicable to explain the effects on microbial communities produced by high environmental UV-B radiation prevailing at the HAAL.

In this work, we present the complete genome sequence of *Acinetobacter* sp. Ver3, highlighting those features providing the molecular basis of its UV-resistome. Furthermore, proteomic profiling of UV-exposed cells was used to determine the genes actually expressed and contributing to this adaptation/resistance mechanisms in a given experimental condition.

## Materials and Methods

### Bacterial Strains, Media, and Culture Conditions

UV-resistant strain *Acinetobacter* sp. Ver3 was previously isolated from Lake Verde (4,400 m asl) at the Andean Puna (Ordoñez et al., 2009) and currently maintained in the culture collection of Laboratory of Microbial Research on Andean Lakes (National System of Biological Data). *Acinetobacter* culture collection strains taxonomically related with Ver3, including *Acinetobacter johnsonnii* DSM 6963, *Acinetobacter lwoffii* DSM 2403, and *Acinetobacter baumannii* DSM 30007 from the German Collection of Microorganisms and Cell Cultures (DSMZ), as well as *Escherichia coli* strains KY1056 and KY1224 were used as controls in the UV-resistance assays (Albarracín et al., 2012). All strains were routinely grown in Luria-Bertani broth (LB, Britania) at 30°C with shaking (200 rpm). Special conditions of preculturing and culturing varied between assays and they are indicated in the corresponding items below. Depending on the resistance, cultures were supplemented with antibiotics at the following final concentrations: tetracycline (Tc), 20 μg/mL; chloramphenicol (Cm), 30 μg/mL.

### UV Assays

UV-B resistance was preliminary tested by a cell survival experiment (Albarracín et al., 2012). Selected strains were grown in LB medium at 30°C with shaking (200 rpm), and cells were harvested in the mid-exponential phase by centrifugation (8,000 rpm, 30 min, 4°C). The pellets were washed twice in 0.9% NaCl and then resuspended in the same solution. 100 μL-aliquots were removed from the tubes, subjected to a dilution series in 0.9% NaCl (10^-1^ to 10^-5^), and 5 μL-aliquots from each were plated on agar plates. The inoculated plates were immediately exposed to UV-B irradiation (09815-06 lamps, Cole Parmer Instrument Company, with major emission line at 312 nm) and duplicates were taken at different time intervals, resulting in increasing doses of UV-B (1–50 kJ m^-2^). During the exposure, UV-B intensity was measured using a 09811-56 radiometer (Cole Parmer Instrument Company) at 312 nm and the tubes covered with acetate sheet to block UV-C. Controls of unexposed samples were run simultaneously in darkness. Growth for each dilution was determined after 48 h of incubation at 30°C in the dark to avoid photoreactivation. For reference, it is worth to mention that non-exposed controls of all strain cultures plated at initial time (T0) reached a mean value of 1 × 10^6^ CFU/ml. Some treatments produced null growth on agar plates (no CFU) even at the lowest dilution (10^-1^) and this was computed in the graphic as zero values. The detection limit was 1 × 10^2^ CFU/ml. Microbial growth was recorded in relation to that of non-exposed cells.

To study the proteomic profile in response to UV-B irradiation, a single pure colony of *Acinetobacter* sp. Ver3 was inoculated in LB broth and incubated at 30°C for 24 h. This preculture was used for further inoculation of 100 ml of LB (initial OD_600__nm_ 0.1) in acrylic tubes until the mid-exponential growth phase (OD_600__nm_ 0.55 ± 0.05) at 30°C. The oxygenation was provided by maintaining a medium culture thickness of 1 cm and the medium in relation 1:5 with the flask. Total dose applied to cultures was 37.4 kJ m^-2^. Parallel dark controls were run in identical conditions, covered with aluminum sheet.

Manipulation of all cultures after UV was made in close room under yellow light (ROSCO supergel medium yellow ROS-010R, ROSCO LABORATORIES, USA) in order to prevent the photoreactivation of cells during dilution and inoculation of samples.

### Statistical Analyses

Statistical analyses were conducted using the Microcal^TM^ Origin Working Model Version 6.0. Paired *t*-test and ONE WAY ANOVA variance analysis were done with a probability level of *p* < 0.05. All experiments were carried out in duplicates.

### Genome Sequencing, Assembly, and Annotation

Genomic DNA from *Acinetobacter* sp. Ver3 strain was purified from cells grown on LB broth for 24 h at 30°C and harvested by centrifugation (3,000 *g* for 10 min at 4°C). The pellets were washed twice with distilled water. Total genomic DNA was extracted with the DNeasy Blood and Tissue Kit (Qiagen) following the manufacturer’s recommendations. Whole-genome shotgun pyrosequencing was performed using a 454 preparation kit (Roche Applied Sciences, Indianapolis, IN, USA) and sequenced with a GS-FLX using Titanium chemistry (454 Life Sciences, Roche Applied Sciences). The 454 reads were assembled with Newbler 2.6. Overall sequence coverage was 23X. The genome sequence of *Acinetobacter* sp. Ver3 was deposited in the NCBI database under accession number JFYL01. Genome annotation was performed using PGAAP from NCBI (Angiuoli et al., 2008) and the RAST annotation server (Aziz et al., 2008; Overbeek et al., 2014).

### Phylogenetic Analysis

Sequence based taxonomic analysis was performed using both 16S rDNA and whole genome comparisons. Sequences from genes and genomes were obtained from IMG database (Markowitz et al., 2014), and are detailed in Supplementary Table S1.

Sequences from 16S rDNA were aligned with Silva Incremental Aligner (SINA; Pruesse et al., 2012) to the rRNA gene databases provided by the SILVA ribosomal RNA project (Quast et al., 2013). Based on this alignment, phylogenetic trees were constructed with Fasttree 2.1.7 (Price et al., 2010) with the Maximum Likelihood method using Jukes Cantor evolution model.

Average nucleotide identity (ANI) analysis of whole-genome data was performed using the method proposed by Goris et al. (2007) implemented in the software JSpecies (Richter and Rosselló-Móra, 2009). Pair-wise ANI values were obtained from BLAST best hits with the script calculate_ANI.py ^1^. A distance matrix to represent the ANI divergence (which is defined as 100% - ANI) between the strains was analyzed with hierarchical clustering by complete linkage method in R, to generate a dendogram.

### Comparative Analysis

All predicted protein sequences were downloaded from GenBank as separate FASTA formatted files. FASTA files were scanned pairwise using the algorithm INPARANOID (Remm et al., 2001; default settings, BLOSUM45 scoring matrix). Unlike most bidirectional best hit-based methods INPARANOID has the advantage that it detects orthologs as well as in-paralogs or co-orthologs (duplicated genes after a recent speciation event). Hence, INPARANOID not only yields a single gene in one genome that is predicted to be the ortholog of a single gene in the other genome (‘single to single’), but also yields ‘many to single,’ ‘single to many’ and ‘many to many’ relationships. Results of pairwise comparisons were analyzed with Multiparanoid (Alexeyenko et al., 2006), generating clusters of orthologous genes. Thus, for each genome a list of clusters could be obtained, and with these lists a Venn graph was generated using jvenn (Bardou et al., 2014). The list does not include genes absent from the clusters, i.e., the unique genes for each strain, which were added to the graph manually. Pseudogenes were not included in the comparison.

### Proteomic Profile of UV-B-Exposed Cultures of *Acinetobacter* sp. Ver3

UV-B exposed cells (as described in point 2.2.) and the corresponding non-exposed controls were harvested by centrifugation (7000 rpm, 10 min) and washed twice with Tris-HCl buffer (0.1 M) pH 7.5. Pellets were resuspended in 5 ml of the same buffer and cells were disrupted in a French press at 2000 psi (SLM Instruments, Inc., Haverhill, MA, USA). Unbroken cells and other debris were removed by centrifugation at 14000 rpm for 10 min at 4°C. Total protein concentration was determined by Bradford method using BioRad reagents (Biorad, Richmond, CA, USA), bovine serum albumin (Sigma) was used as standard. Aliquots of 400 μg were stored at -80°C until the isoelectrofocusing assay. Three independent assays for each condition were performed.

### Two-Dimensional Electrophoresis (2DE)

Sample preparation and two-dimensional electrophoresis (2DE) gels were carried out according to Sánchez et al. (2005) with some modifications. To remove nucleic acids, samples containing 400 μg of proteins were treated with 1 μl of bezonase (Novagen^®^) in presence of 1 μl of 1 M MgSO_4_ for 30 min at 37°C. Total proteins were precipitated with three volumes of cold acetone, after incubation at -20°C for 16 h, samples were centrifuged (14000 rpm, 10 min). The protein pellets were air-dried and solubilized in 40 μl of solubilization mixture. The suspension was centrifuged at 3500 rpm for 10 min and loaded onto immobilized pH gradient strips (pH 4.0 to 7.0, 18 cm, GE Health Care, Sweden). Gels were passively rehydrated for 20 h. The isoelectrofocusing assay was performed using IPGphor (GE HealthCare, Sweden) at 53,500 V/h and the focusing strips were stored at -20°C until second dimension was performed. The second dimension was performed by SDS-PAGE on gels containing 12.5% (w/v) polyacrylamide, carried out in a Bio-Rad Protean II xi cell (Biorad, Richmond, CA, USA), and proteins were resolved overnight at a constant current of 11 mA/gel at 4°C. Gels were stained with Biosafe colloidal Coomassie blue (Biorad, Richmond, CA, USA) according to the manufacturer’s instructions, scanned with Image Scanner III and analyzed with Prodigy SameSpots (Nonlinear Dynamic Group, UK).

### Protein Identification using Peptide Mass Fingerprinting

For each independent assay, the respective 2-DE gels were analyzed as described before (Belfiore et al., 2013). Prominent spots were used to manually assign vectors in each gel image and the automatic vectors feature of the software was used to add additional vectors, which were manually verified. These vectors were used to warp the images and align the spot position to a common reference gel. Spot detection performed according to this reference gel was edited and artifacts removed. To correct the variability due to staining and to reflect the quantitative variation in intensity of protein spots, the spot volumes were normalized as a percentage of the total volume in all spots in the gel. A spot was considered significant when its resulting normalized volume showed more than 1.2 fold variation with respect to the control (LB) at the level of *p* < 0.05.

Individual spots were excised from gels and the mass spectrometry analyses were carried out by CEQUIBIEM (Centro de Estudios Químicos y Biológicos de Espectrometría de Masa), Facultad de Ciencias Exactas y Naturales, UBA, Argentina. To determine proteins identity, MASCOT (Matrix Science Inc., Boston, MA, USA ^2^) program was used to identify proteins from peptide mass fingerprints. Fragmentation was carried out with more intense MS peaks (MS/MS). When possible, MS and MS/MS information was combined for one or more peptide searches. MASCOT search engine (Matrix Science Inc., Boston, MA, USA^2^) was used to identify proteins from peptide mass fingerprint data.

## Results and Discussion

### Taxonomic Affiliation of *Acinetobacter* sp. Ver3

**FIGURE 1:**
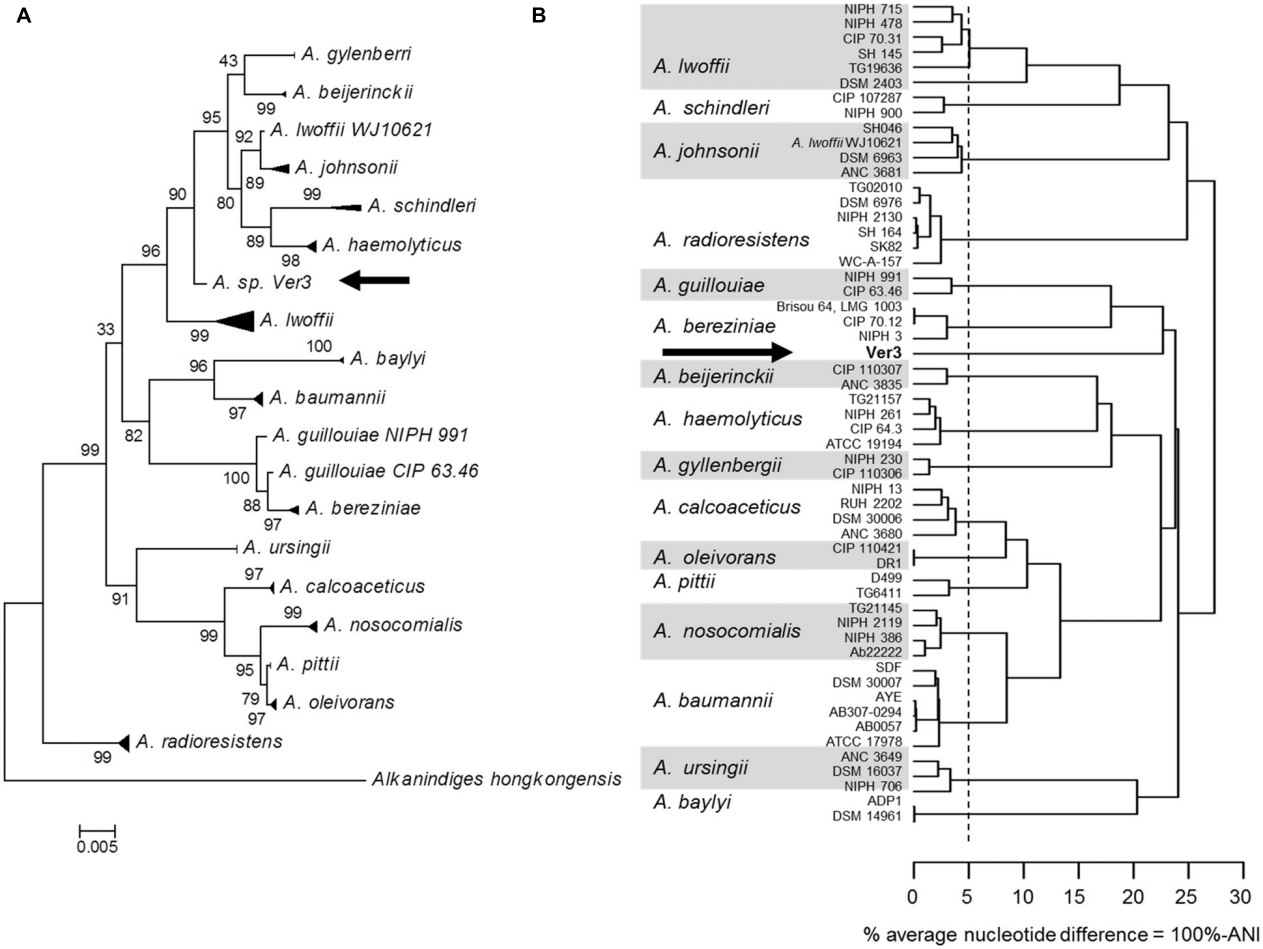
Phylogenetic analysis of 55 *Acinetobacter* strains. Strain *Acinetobacter* sp. Ver3 is indicated with an arrow. **(A)** Maximum likelihood tree from 16S rRNA gene. **(B)** Dendogram based on whole genome average nucleotide identity (ANI). The vertical dashed line represents the proposed 95% species cutoff (Goris et al., 2007).

*Acinetobacter* sp. Ver3 was first isolated by Flores et al. (2009), and initially assigned to the genus by partial 16S rDNA sequencing. Further analysis compared the strain to other members of the genus and suggested that it would form a different clade than other known species, such as *A. baumanii* or *A. lwofii* (Di Capua et al., 2011). The full 16S rDNA sequence from Ver3 showed 98.83% identity with *A. lwoffii* WJ10621 and 98.76% identity with both *A. johnsonii* strain ATCC 17909 (also known as CIP 64.6) and *A. beijerinckii* CIP 110307. A phylogenetic tree based on this marker also suggests a close relationship with the groups of *A. johnsonii* and *A. lwoffii* (**Figure 1A**). However, whole genome analysis using the ANI method shows different results (**Figure 1B**). In this analysis Ver3 does not group with these species, and has a lower percent average nucleotide difference (defined as 100% - ANI) value than the proposed 95% threshold (Richter and Rosselló-Móra, 2009) suggesting that it could be a novel species.

### UV-Resistance Profile of Ver3 and Comparison with Reference Strains

**FIGURE 2:**
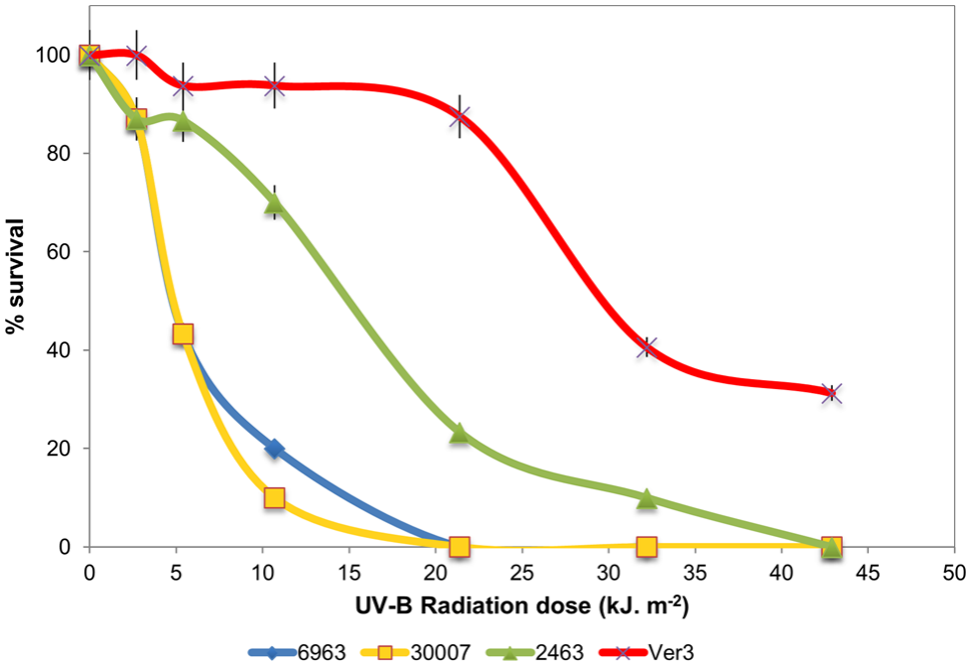
**Survival of strains *A. baumannii* DSM 30007, *A. johnsonii* DSM 6963, *A. lwoffii* DSM 2403, and *Acinetobacter* sp. Ver3 under UV-B exposure**.

Strain Ver3 was able to grow at higher doses than its closely related taxonomic neighbors: *A. lwoffii* DSM 2403, *A. johnsonii* DSM 6963 and *A. baumannii* DSM 30007 (**Figure 2**). Even after the highest dose tested (ca. 43 kJ m^-2^), Ver3 remained viable (30% survival) while CFU remained below detection limit for all other *Acinetobacter* strains. These results confirmed previous observations (Di Capua et al., 2011) and indicate that UV-resistance for Ver3 appeared more as a consequence of its original environmental adaptive pressure than of its phylogenetic affiliation. For comparison, in *E. coli* KY1056, a photolyase-containing UV tolerant strain and its knock-out mutant, *E. coli* KY1225, the lethal dose (1.2 kJ m^-2^) was very much lower than that for the *Acinetobacter* spp. (Albarracín et al., 2014). In exponential phase, *Deinococcus radiodurans* is 33-fold more resistant to UV than is *E. coli*, but these results are considered for UV-C (Sweet and Moseley, 1974). Bauermeister et al. (2009) exposed *D. radiodurans* wild-type strain R1 and the mutant strains deficient in *recA, pprA,* and *irrE* to monochromatic UV-C (254 nm) and polychromatic UV-(A + B; >280 nm) and UV-A (>315 nm) radiation. Under polychromatic UVA+B, the wild-type strain demonstrated a 10% survival after 52.3 kJ m^-2^ while the mutants *pprA, irrE,* and *recA* were sensitive at lower doses; i.e., 10% survival was observed with UV-doses of 44.2, 33.9, and 37.8 kJ m^-2^, respectively (Bauermeister et al., 2009).

### General Genome Features

The genome of *Acinetobacter* sp. Ver3 consists of 3,345,299 bp, with a GC content of 38.9%. NCBI PGAP annotation shows 3,241 genes, including 3,080 coding sequences, 66 RNAs and 95 pseudogenes. These data is shown in **Table 1**, and compared to the *Acinetobacter* strains with data on their UV-resistance profiles (**Figure 2**).

**Table 1:** General features of the genomes of the *Acinetobacter* strains used in this study.

	Ver3	*A. baumannii* DSM 30007	*A. johnsonii* DSM 6963	*A. lwoffii* DSM 2403
Genome size (bp)	3,345,299	3,971,516	3,582,699	3,206,079
GC content (%)	38.9	38.7	41.5	43.0
Contig count	209	22	48	29
CDS count	3175	3766	3488	3106
RNA count	66	66	102	87
Pseudo genes count	95	0	1	0
Genes in RAST subsystems	1445	1784	1511	1394

**FIGURE 3:**
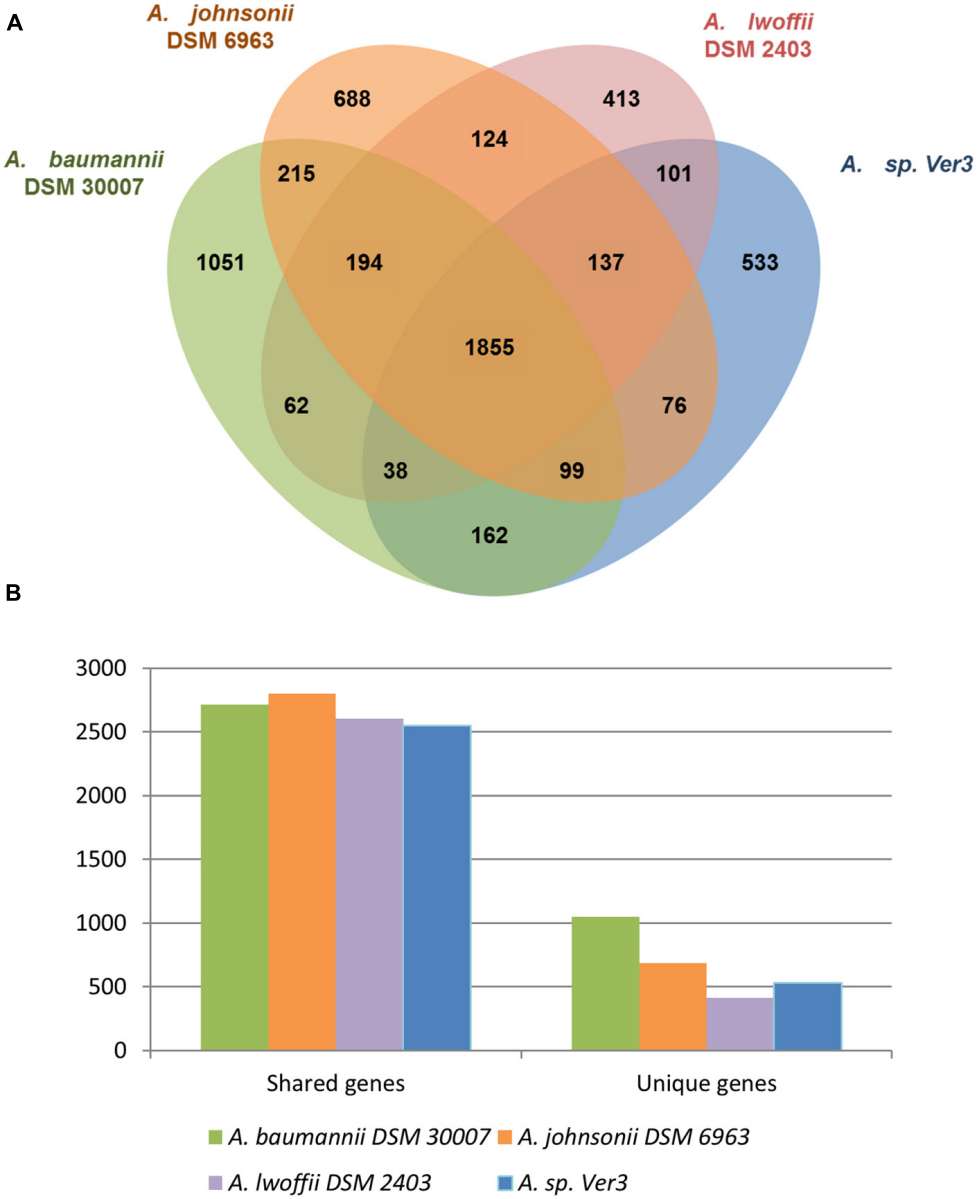
Genome comparison of the strains *A. baumannii* DSM 30007, *A. johnsonii* DSM 6963, *A. lwoffii* DSM 2403, and *Acinetobacter* sp. Ver3. **(A)** Orthologous coding sequences were clustered and the distribution is shown as a Venn graph. **(B)** Number of unique and shared genes.

RAST annotation server (Aziz et al., 2008; Overbeek et al., 2014) was used for a broad view of genome features. 1445 genes (46.91%) from Ver3 were included in the Subsytems classification, revealing some of the polyextremophilic features of this organism, such as genes for resistance to UV irradiation, metals and metaloids, salinity, and even antibiotics. At a broad level, this classification did not show remarkable differences between the four strains. They were further pairwise compared based on protein sequences (see Materials and Methods), which generated clusters of orthologous genes, as shown on **Figure 3**. The numbers in the Venn diagram represent clusters, which might contain more than one gene from a single species, but roughly more than 60% of the genes are shared among all the strains. This methodology allowed detecting strain specific genes, 17.3% of the total in Ver3 case, which are detailed in Supplementary Table S2. These unique genes would confer differential capabilities, and were further analyzed in the following sections.

### Genomic Data Related with *Acinetobacter* sp. Ver3 UV-Resistome

UV-resistance is a complex trait that might arise from a battery of genes dedicated to avoid or alleviate the damage provoked by UV. As this damage is due to direct causes, such as the effects of the radiation itself on DNA, or to indirect causes such as the generation of ROS, the full set of genes involved in this phenotype is hard to define. Nevertheless, in a constantly UV-exposed environment (i.e., the HAAL), a complex system of genetic and physiological mechanisms (UV-resistome) is expected to have evolved and be shared by the indigenous microbial community, including Ver3 strain. Following this asumption, HAAL’s UV-resistome shall comprise several or all of the following sub-systems: (i) highly sensitive sensing component to the external detrimental stimuli (UV-photoreceptor or other stress sensor) and effective response-regulators; (ii) UV shields such as special cell envelope and/or UV-absorbing pigments; (iii) an enhanced capacity for scavenging the reactive molecular species responsible for oxidative damage; (iv) an extremely efficiently use of conventional set of DNA repair proteins; (v) tolerance systems to DNA (i.e., error prone polymerases), protein and lipid damages. In the following sections, the genome description of Ver3 focuses on three of these hypothetic subsystems: DNA repair and response to oxidative damage as experimental evidence previously indicated their involvement in Ver3 UV-resistome (Di Capua et al., 2011; Albarracín et al., 2012, 2014), and putative regulators.

#### DNA Repair Mechanisms

The pathways present in Ver3 related to UV damage repair, including direct damage and indirect oxidation damage (Dalhus et al., 2009) are outlined in **Table 2** and addressed below. Three genes were unique to Ver3 (highlighted in **Table 2**).

**Table 2:** DNA repair genes found in *Acinetobacter* sp. Ver3.

Repair system	Gene	NCBI accession	Function
**Excision repair**
Base excision repair (BER)	*mug^∗^*	EZQ06622	G:T/U mismatch-specific DNA-glycosylase
	*ung*	EZQ12129	Uracyl-DNA glycosylase
	*ndk*	EZQ11607	AP endonuclease
	*mutM*	EZQ01011	(8-oxoG) DNA glycosylase
	*mutY*	EZQ12003	A/G-specific adenine glycosylase
	*nth*	EZQ02198	5′ AP endonuclease – endonuclease III
	*exoIII*	EZQ11928	Exonuclease III
	*tag*	EZQ12006	Methyl-A glycosylase I
Nucleotide excision repair (NER)	*uvrA*	EZQ11284	DNA binding
	*uvrB*	EZQ11685	Helicase – 3′ incision endonuclease
	*uvrC*	EZQ10970	5′ Incision endonuclease
	*uvrD*	EZQ10386	Excision helicase
	*mfd*	EZQ12414	Transcription- repair coupling factor
Mismatch repair (MMR)	*mutL*	EZQ12151	Binds MutS
	*mutS*	EZQ10668	Binds mismatches and loops
**Recombinational repair**
**Non-homologous end-joining (NHEJ)**	**Not present**	
Homologous recombination		
*Initiation- RecBCD pathway*	*recA*	EZQ12424	Recombinase
	*recB*	EZQ12371	Exo V helicase
	*recC*	EZQ12370	Exo V nuclease
	*recD*	EZQ12372	Exo V helicase
*Initiation- RecFOR pathway*	*recF*	EZQ02163	Assist RecA filamentation
	*recO*	EZQ10098	Binds ssDNA, assists RecF
	*recR*	EZQ10461	ATP-binding, assists RecF
	*recN*	EZQ10978	ATP-binding
	*recJ*	EZQ11310	5′–3′ ssDNA exonuclease
	*recQ*	Not present	ATP-dependent DNA helicase
*Initiation- SbcBCD pathway*	*sbcB*	Not present	
	*sbcC*	EZQ09958	Hypothetical protein CL42_09425
	*sbcD*	EZQ09959	ATP-dependent dsDNA exonuclease
	*ssb*	EZQ00997	Single strand binding protein
*Branch migration*	*ruvA*	EZQ11507	5′–3′ junction helicase (with RuvB)
	*ruvB*	EZQ11506	5′–3′ junction helicase (with RuvA)
*Resolvases*	*rucC*	EZQ12511	Junction endonuclease
	*recG*	EZQ11853	Resolvase, 3′–5′ junction helicase
**Other systems**
SOS and error prone repair 3 *umuDC* operons	*lexA*	Not present	SOS activator
	*umuC*	EZQ06645	Error prone DNA polymerase V subunit C
	*umuD*	EZQ06644	Error prone DNA polymerase V subunit D
	*umuC*	EZQ01410	Error prone DNA polymerase V subunit C
	*umuD*	EZQ01411	Error prone DNA polymerase V subunit D
	*umuC*	EZQ01153	Error prone DNA polymerase V subunit C
	*umuD*	EZQ01152	Error prone DNA polymerase V subunit D
	*ddR*	Not present	Regulator in *A. baylyi*
	*dinB*	EZQ03773	Error prone DNA polymerase IV
Alkyltransferases	*ogt*	EZQ02427	Alkyltransferase with regulatory motif
Photolyases	*phr*	EZQ09984	Deoxyribodipyrimidine photolyase
	*phlK*	EZQ01671	Photolyase-like

*Comparison with strains *A. baumannii* DSM 30007, *A. johnsonii* DSM 6963, and *A. lwoffii* DSM 2403 showed that the highlighted genes are present only in Ver3*.

*^∗^Not homologous to *E. coli mug*, but belongs to the same family*.

##### Excision repair

###### Base excision repair

The BER system repairs oxidative damage to DNA (a likely consequence of UV), by action of glycosylases that remove damaged bases leaving an AP (apurinic/apyrimidinic) site, and endonucleases that bind to this site and cleave the DNA 5′ to the abasic site, forming a free 3′-hydroxyl which is repaired by DNA polymerases (Dalhus et al., 2009). Ver3 encodes a number of glycosylases related to this system (**Table 2**), including both monofunctional (Ung) and bifunctional (MutM, Nth) enzymes (Saito et al., 1997), in addition to the AP exonuclease III (Souza et al., 2006). The Ndk protein, reported to act as a partner for Ung in *E.coli* (Goswami et al., 2006), is also present.

###### Nucleotide excision repair (NER)

The nucleotide excision repair (NER) system identifies bulky distortions on the DNA strand and removes the damaged single strand region, which is repaired by DNA polymerase I (Truglio et al., 2006). In Ver3, all of its components, including UvrA and UvrB that recognize the lesion, the UvrC nuclease and the UvrD helicase that remove the damaged strand are present. A Mfd protein homolog is also present, which might recruit the machinery to the lesion (Savery, 2007) when RNA polymerase stalls, coupling repair to transcription. Homolog uvrA, uvrB, and uvrC genes were also important for the configuration of UV-resistance of *Halobacterium*. Deletion of these genes resulted in UV-hypersensitive cells in conditions where photoreactivation light was absent (Crowley et al., 2006).

###### Mismatch repair (MMR)

DNA mismatches may be caused by replication errors, or more likely, base damage. The mismatch repair (MMR) system recognizes deformations in the helix due to this, and acts upon the newly synthesized strand (Dalhus et al., 2009). MutL and MutS are present in Ver3, and also in *Acinetobacter baylyi* ADP1, where they have been shown to be involved in mismatch recognition to prevent recombination of foreign DNA (Young and Ornston, 2001). Homologs to the MutH endonuclease were not found, not only in Ver3, but also in none of the sequenced *Acinetobacter* in NCBI. Absence of MutH has also been reported in *Pseudomonas* (Oliver et al., 2002) so another yet unidentified protein might be fulfilling MutH role in these genera.

##### Recombinational repair–double strand repair pathways

###### Non-homologous end-joining (NHEJ)

This pathway is absent from Ver3, at least no homologs were found in our strain to the eukaryotic DNA-end-binding protein, Ku, and DNA ligase IV, as identified in several bacteria (Weller et al., 2002).

###### Homologous recombination (HR)

Homologous recombination (HR) is a ubiquitous process, crucial for DNA repair and maintenance. In Ver3, many genes are related to HR, including most homologs to known *E. coli* genes (**Table 2**). In *Acinetobacter*, it also has a role in the acquisition of foreign DNA (de Vries and Wackernagel, 2002). RecA is the central protein, and in *A. baumannii* is involved in cellular protection against stresses induced by DNA damaging agents, several classes of antibiotics, and oxidative agents (Aranda et al., 2011). The HR process can be divided into four main steps: (a) initiation (generation of recombination substrate); (b) strand pairing and exchange; (c) branch migration and (d) branch resolution (Eisen and Hanawalt, 1999). Even though several initiation pathways are known, it has been suggested that only the RecBCD pathway is functional in *A. baylyi* ADP1, as double mutants *recBCD recJ* are not viable (Kickstein et al., 2007), and a similar situation might arise in Ver3.

##### Other DNA repair systems

###### SOS response, error prone repair, and Y type polymerases

Ver3 genome encodes three operons containing error prone DNA polymerase type V components (*umuDC*), and also a type IV DNA polymerase homolog. This might suggest an SOS mutagenic response to DNA damaging agents, as observed in *E. coli* and other bacteria (Courcelle et al., 2001). However, the response is quite different in *Acinetobacter*, particularly because mutagenesis is rarely seen, with the notable exception of the emerging pathogens *A. baumannii* and *Acinetobacter ursingii.* This is intriguing as most species present two to four copies of the *umuDC* locus, each copy belonging to a different class (Hare et al., 2012). A group of *Acinetobacter* UmuD proteins are 1.5 times larger than the *E. coli* counterparts, being named UmuDAb (Hare et al., 2006). Studies on *A. bayly* ADP1 and *A. baumanii* ATCC 17978 showed that two *umuDAb* genes, ACIAD2729 and A1S_1389 respectively, would have a similar function to *E. coli* LexA (Aranda et al., 2014; Hare et al., 2014). The proteins share 80% identity, but the closest Ver3 BLAST best hit has only 55% identity. Also, all Ver3 *umuD* genes are upstream of an *umuC* gene, whereas only an *umuC* fragment is found downstream ACIAD2729, and no *umuC* downstream A1S_1389. Thus, the presence of a homolog in Ver3 is not clear, suggesting perhaps a different regulation. Our comparative analysis classifies the three different Ver3 UmuD and UmuC proteins in separate orthologous groups, being one UmuC and one UmuD proteins unique from Ver3. Thus they might have different functions, i.e., bypassing different lessons, as proposed in other studies in *A. baumanii* ATCC 17978 (Norton et al., 2013). This work found that multiple DNA polymerase V gene components are all expressed at different levels and induced upon DNA damage. Different DNA-damaging agents caused distinct expression of *umuD* and *umuC* genes, indicating perhaps different lesion-bypass abilities. DNA polymerase IV upregulation was observed only at the protein level.

UVB-induced (1.2 kJ m^-2^) DNA photodamage previously evaluated in *E. coli* reference strains (Albarracín et al., 2014) indicated that an average value of 200 cyclobutane pyrimidine dimers (CPDs) per 10^6^ bases was deleterious for mid-log phase *E. coli* strains. Interestingly, a similar dose applied to Ver3 cells in the same growth phase produce a bigger amount of damage (ca. 400 CPDs per 10^6^ bases) while viable population was maintained unchanged respect to the non-exposed control. Evidently, Ver3 UV-resistome includes a mechanism for high tolerance to accumulation of DNA lesions and by-pass for DNA replication, which would also explain the extensive battery of genes related to translesion replication (**Table 2**).

###### Photolyases and related proteins

Two genes encoding proteins from the photolyase family are present in Ver3. The proteins belong to different subfamilies, with distinct functions. One of them is a bona fide Class I CPD-photolyase, monomeric flavoproteins that contain two non-covalently bound chromophore cofactors: flavin adenine dinucleotide (FAD; also known as the catalytic cofactor) and usually methenyltetrahydrofolate or 8-hydroxy-7,8-didemethyl-5-deazariboflavin (8-HDF; the antenna pigment; Sancar, 2003). They repair DNA damage specifically produced by UV such as CPDs and pyrimidine (6–4) pyrimidone photoproducts (6-4PPs) at dipyrimidine sites, where two pyrimidine (Py) bases are juxtaposed in tandem in the nucleotide sequence of DNA (Ikehata and Ono, 2011). The function of the Ver3 protein was confirmed in an heterologous host (Albarracín et al., 2014). Recent work on HAAL environmental strains examined accumulation of CPDs and 6-4PPs in *Acinetobacter* UV-resistant strains N40, Ver3, Ver5, and Ver7, compared to type strains *A. johnsonii* DSM 6963 (AJ) and *A. baumannii* DSM 30007 (AB). Although HAAL strains displayed higher UV-B resistance profiles, they also showed the highest number of photoproducts, roughly 25% more photoproducts than the controls. A DNA lesion bypass without DNA damage repair could again explain this pattern of high survival plus high photoproducts accumulation performed by the several type V DNA polymerases found in Ver3 genome (**Table 2**). Likewise, photorepair in HAAL strains was more effective than in the controls AB and AJ and always more effective than dark repair (Albarracín et al., 2012), suggesting that photolyases would be very important to repair these lesions.

The second photolyase-like protein in Ver3 belongs to a newly characterized group of the cryptochrome-photolyase family (CPF), called as iron–sulfur bacterial cryptochromes and photolyases (FeS-BCP). Even though the protein is “unique” in Ver3 when comparing four strains on this work, a BLAST search against NCBI nr database filtered for different *Acinetobacter* species retrieved proteins with high identity (>90%) in other *A. baumannii* and *A. johnsonii* strains, but no homologs were found in *A. lwoffii.* This kind of proteins display a Fe–S cluster and a new chromophore 6,7-dimethyl-8-ribityllumazine (DMRL) being widespread in most available prokaryote genomes (Oberpichler et al., 2011; Zhang et al., 2013). Ver3 protein PhlK (EZQ01671) has 50.4 and 49.2% identity to RsCryB and PhrB, respectively, the only two members of this family that have been characterized. PhrB is found in *Agrobacterium tumefaciens* (Oberpichler et al., 2011; Zhang et al., 2013) and RsCryB in *Rhodobacter sphaeroides* (Geisselbrecht et al., 2012). No photorepair activity was observed *in vitro* for RsCryB, but it was proved to control the expression of genes of the photosynthetic apparatus. Instead, PhrB was able to remove 6-4PPs, constituting the first report of a prokaryotic (6–4) photolyase. The function of Ver3 protein is difficult to assess by direct comparison, but it might be involved in response to light stimuli. It was reported that [4Fe–4S] cluster of RsCryB can readily be oxidized, and thus RsCryB might act as sensor for ROS upon photooxidative stress (Geisselbrecht et al., 2012). A likely role in Ver3 PhlK on sensing the photooxidative UV-induced stress for rapidly reacting in DNA repair may be another feature given Ver3 UV-fitness. Current work is directed to obtain knock-mutants on this gene in Ver3 to help elucidating its cryptic function. *Halobacterium* sp. NRC1, another radiation resistant organism, also bears two photolyase genes, and only one was proved to act as a CPD photolyase by analysis of knockout mutants. However, the second gene encodes a protein of the CPD photolyase family, and thus its function is intriguing (Baliga et al., 2004).

In sum, the superior photorepair activity observed in Ver3 could be due to a very efficient activity of one or both proteins. Not only that, but also the photolyases might also be enhancing other DNA repair systems (Albarracín et al., 2014).

#### Oxidative Stress Response

Reactive oxygen species are unavoidable by-products of aerobic life, being the electron transport chains one of their main sources (Starkov, 2008). Two different processes, generation and degradation of ROS, usually are under delicate cellular control keeping very low steady-state ROS concentrations. Additional sources of ROS can affect this balance leading to a pro-oxidant condition named-to as “oxidative stress.” For example, exposure to environmental hazards, such as high toxic metaloids or UV radiation, can damage the cell indirectly by promoting oxidative stress (Lushchak, 2011). Oxidative stress response has been shown to vary among bacteria and to affect directly and indirectly a good number of cellular processes (Chiang and Schellhorn, 2012). The generated ROS lead to oxidative damage of cell components as membrane lipids, nucleic acids and proteins (Shiu and Lee, 2005; Li et al., 2010). Living organisms have developed antioxidant strategies to cope with ROS accumulation, including the evolution of enzymatic scavengers such as catalase and superoxide dismutase (SOD), which represent two conspicuous antioxidant enzymes widely distributed in nature (Sies, 1997). Herein, we analyzed the presence of conserved regulation machinery and some of the possible response genes directly involved in ROS degradation in Ver3. The genes found are listed in **Table 3**.

**Table 3:** Genes related to oxidative response found in *Acinetobacter* sp. Ver3.

Oxidative response	NCBI accession	Function
**Regulators**
*oxyR*	EZQ10419	H_2_O_2_ induced regulator
*soxR*	EZQ11770	O_2_^-^ induced regulator
*soxS*	Not found	
*furR*	EZQ09953	Fe induced regulator
**SODs**		
*sodB*	EZQ10255	Cytoplasmic FeSOD
*sodC*	EZQ12222	Periplasmic CuZnSOD
**Catalases and peroxidases**	
*kat*	EZQ12194	Cytoplasmic catalase
	EZQ11977	Periplasmic catalase
*ahpC1* (two paralogs)	EZQ07158	Alkylhydroperoxidase C
	EZQ01175	Alkylhydroperoxidase C
	EZQ10124	Thiol peroxidase BCP type
	EZQ03014	Glutathione peroxidase
	EZQ12479	Putative peroxidase
	EZQ04963	Rubrerythrin
*ohr*	EZQ02177	Organic hydroperoxide resistance protein
	EZQ10931	Glutathione peroxidase
**Damage to non-DNA molecules**	
Lipids and membranes	EZQ03777	Lysophospholipase
	EZQ10901	Lysophospholipase
	EZQ12493	Lysophospholipase
	EZQ10434	Glycerophosphoryl diester phosphodiesterase
	EZQ10435	Glycerophosphoryl diester phosphodiesterase
	EZQ11565	Glycerophosphoryl diester phosphodiesterase
	EZQ06623	Lysophospholipase L1 and related esterases
	EZQ10413	Lysophospholipase L1 and related esterases
Proteins	EZQ10372	Peptide methionine sulfoxide reductase
	EZQ10932	Conserved domain frequently associated with peptide methionine sulfoxide reductase

*Comparison with strains *A. baumannii* DSM 30007, *A. johnsonii* DSM 6963, and *A. lwoffii* DSM 2403 showed that highlighted genes are either unique in Ver3, or present in only one of the other strains*.

##### Regulators

Bacteria respond to oxidative stress by modulation of a number of genes. This is achieved through the action of regulators. Homologs to the best known regulators OxyR, SoxR, and Fur are found in Ver3 (**Table 3**). *oxyR* has been identified in *A. bayly* ADP1 and Ver3 has the same genetic organization (Geissdörfer et al., 1999). The *oxyR* regulons respond to hydrogen peroxide induced stress by activating the expression of detoxification enzymes such as catalases and hydroperoxidases, among others (Lushchak, 2011). SoxR and Fur have less conserved regulons (Chiang and Schellhorn, 2012). Conservation of the SoxR system is limited to enterobacteria and SoxR homologs from *Pseudomonas* and *Streptomyces* have been shown to be related to quorum sensing (QS) and secondary metabolite transport rather than response to superoxide anion (Dietrich et al., 2008). Likely, a similar situation might occur in Ver3. Finally, Fur from Ver3 is 91% identical to the protein from *A. baumannii* ATCC 19606, which has been shown to control the transcription of iron regulated genes (Nwugo et al., 2011). The Fur repressor is the principal regulator of iron homeostasis in *E. coli,* which is coordinated with responses to oxidative stress (Cornelis et al., 2011).

##### Catalases and peroxidases

A number of proteins are annotated in Ver3 as catalases and peroxidases (**Table 3**). These enzymes control the degradation of H_2_O_2_ and other organic peroxides. Catalases and NADH peroxidase (Ahp) are primary scavengers in many bacteria, whose activities have been unambiguously demonstrated. A wide variety of additional enzymes have been proposed to serve similar roles as observed in *in vitro* assays, but their contributions *in vivo* remain unclear (Mishra and Imlay, 2012).

Catalases differ from peroxidases in that they do not require reductive cofactors (Zamocky et al., 2012) Genome sequence shows two catalase genes in Ver3, and one of them (EZQ11977) is predicted to have a signal peptide by SignalP (Petersen et al., 2011) which would send it to periplasm, according to PSORT prediction implemented in RAST server (Aziz et al., 2008; Overbeek et al., 2014). Our comparative analysis showed a total of 11 genes annotated as catalases in the selected *Acinetobacter* strains: two in Ver3, four in *A. baumannii*, four in *A. lwoffii*, and one in *A. johnsonii*. These catalases were classified in five clusters of orthologs: (i) Ver3 EZQ12194 groups with a protein from *A. lwoffi*, sharing 92.5% sequence identity; (ii) orthologs to Ver3 EZQ11977 are found in *A. baumannii* and *A. lwoffii*, also with predicted signal peptides, (iii) another cluster groups proteins from *A. baumannii*, *A. lwoffii*, and *A. johnsonii*, (iv) another cluster has one member from each *A. baumannii* and *A. lwoffii*; (v) finally, one last protein from *A. baumannii* does not group with any other. From the genomic background, catalase by itself might not be determinant of the enhanced resistance of Ver3 to UV, as all of the organisms have catalases. However, previous work found a relationship between catalase activity and UV-B resistance (Di Capua et al., 2011). Two active catalase species were present in most *Acinetobacter* strains tested, while a single catalase band was visible in Ver3 that would correspond to the cytoplasmic catalase EZQ12194, and no bands were detected in *A. johnsonii*. Catalase activity measurements in soluble extracts were much higher for Ver3 than for any control strain, suggesting that this enhanced activity would be responsible for the phenotype. Likely, expression levels would be important for resistance. The role of the periplasmic catalases is not clear. They have been proposed as defense against host microbicidal mechanisms. Periplasmic catalases have been found in *E. coli* O157: H7 (Brunder et al., 1996), *Brucella abortus* (Sha et al., 1994), *Pseudomonas syringae* (Klotz and Hutcheson, 1992), *Vibrio fischeri* (Visick and Ruby, 1998) and *Legionella pneumophila* (Amemura-Maekawa, 1999), all of them organisms involved in colonization of an eukaryotic host.

Peroxidases fall into two categories: thiol-based peroxidases and non-thiol peroxidases, depending on the active site chemistry (Mishra and Imlay, 2012). Ver3 has several genes for both types of proteins, including: alkylhydroperoxide reductases (AhpCF), thiol peroxidases (Tpx), bacterioferritin comigratory protein (BCP), glutathione peroxidase (Gpx), and organic hydroperoxide resistant protein (Ohr). Non-thiol peroxidases include rubrerythrin. Homologs from all of these proteins have been shown to degrade peroxides *in vitro* in other systems, but their *in vivo* function is not confirmed. There are two copies of the *ahpC* gene, which is usually associated with *ahpF*, encoding the AhpC reductase (Jacobson et al., 1989). This association is not observed in Ver3, bearing also two copies of *ahpF*, but in different locations away from *ahpC*. Interestingly, Ver3 AhpFs are smaller than other characterized proteins, and have only one domain with homology to C-terminal *Salmonella typhimurium* AhpF. Both *ahpF* genes are in the very 5′ end of the contigs, the actual ORFs might be longer, and thus we cannot confirm their size from this data, although short genes annotated as *ahpF* can be found in *Acinetobacter* strains but have never been characterized. Given the genomic location and the unusual size of AhpFs, whether they actually work as AhpC reducing pairs is not clear. As AhpC homologs have been definitely implicated in H_2_O_2_ resistance (Mishra and Imlay, 2012), the presence of two copies in Ver3 might be related to its enhanced resistance. The *Acinetobacter* Ver3 Ohr putative protein has a high identity with the protein from *Pseudomonas aeruginosa* (71.7%) and bears the two conserved Cys required for functionality (Lesniak et al., 2002). Studies on *Xylella fastidiosa*, *Xanthomonas campestris*, *A. tumefaciens,* and *P. aeruginosa ohr* mutants show normal resistance to H_2_O_2_ but unusual sensitivity to *t*-butyl hydroperoxide and cumene hydroperoxide (Mongkolsuk et al., 1998; Ochsner et al., 2001; Cussiol et al., 2003; Chuchue et al., 2006). This genetic evidence strongly supports the idea that Ohr is the primary scavenger of organic peroxides in these organisms.

##### Superoxide dismutases

Superoxide anion is another ROS. Although the O_2_^-^ and H_2_O_2_ stress responses are distinct, the conditions are related, via the chemical conversion of O_2_^-^ to H_2_O_2_ by SODs. There are four known metalloforms of SOD, identified by their metal centers: Fe, Mn, CuZn, and Ni (Wolfe-Simon et al., 2005). *Acinetobacter* sp. Ver3 encodes a predicted cytoplasmic FeSOD (EZQ10255) and a periplasmic CuZnSOD (EZQ12222). Experimental results support the presence of the FeSOD in strains previously studied, the metal identity was assessed by selective inhibition with H_2_O_2_ (Di Capua et al., 2011). These enzymes have different primary and tertiary structures and almost certainly evolved independently. FeSOD or MnSOD are usually essential in aerobic organisms, the presence of a cytoplasmic CuSOD in the periplasm of α, β, and γ proteobacteria is intriguing (Wolfe-Simon et al., 2005). For pathogenic organisms, it has been suggested that the enzyme might have a role in resistance to exogenously produced O_2_^-^, which could readily enter cells at the acidic phagosomal pH as the protonated uncharged form (Gort et al., 1999; Korshunov and Imlay, 2002). However, the enzyme is also present in non-pathogenic bacteria, and here the source of external O_2_^-^ is more difficult to identify. In *Caulobacter crescentus* it has been hypothesized that an environment with plant-derived polyphenols and high oxygen due to algal photosynthesis, such as a pond, might have the conditions to generate harmful concentrations of O_2_^-^ (Schnell and Steinman, 1995). Both scenarios might apply to Ver3. It was isolated from Lake Verde, home to flamingos, which Ver3 could infect as an opportunistic pathogen. Also, microbial mats are present on the pond, where cyanobacteria could be producing high oxygen concentrations.

##### Damage to non-DNA molecules

Several proteins known to repair oxidative damages to proteins or participate in membrane homeostasis under oxidative stress (Ghosal et al., 2005) can be found in Ver3 (**Table 3**). Unspecific (non-DNA targeted) damage has been considered as a major cause of cell death exposed to radiation (Daly, 2012; Krisko and Radman, 2013). This mostly refers to high-energy radiation, such as gamma rays or UV-C. However, UV-A (350 nm) can also kill bacteria, suggesting that radiation-generated oxidative damage to non-DNA molecules can also be lethal. The mechanisms underlying the process are not clear, neither the resistance nor repair machinery (Bosshard et al., 2010). An unspecific mechanism to control oxidative damage include the presence of low Fe^2+^/Mn^2+^ ratios in *Deinococcus* and other species (Aguirre and Culotta, 2012), but this system was not assessed in Ver3.

#### Unique Regulators

Given the unique high UV exposed environment, possible UV sensors with a function similar to plant UVR8 (Fraikin et al., 2013) were searched. Among the unique putative regulators from Ver3, 23 regulators were found, but there is no evidence that any of them is involved in UV-resistance, in fact several of them have metal binding sites, suggesting that they might be related to resistance to other environmental stress. One protein (EZQ10399) has two PAS domains including heme pockets (NCBI Conserved Domains Database accession cd00130), which might be involved in oxygen/redox sensing. It also bears histidine kinase and ATPase domains, and the neighboring gene, also unique, would be a DNA-binding response regulator belonging to LuxR family (EZQ10400). Even though these latter proteins might have a role in oxidative stress, none of these regulators seems to be directly involved in UV detection, at least none of them shows any known domain possibly involved in this function.

### Differential Proteome of UV-Exposed *Acinetobacter* sp. Ver3 Cell Cultures

The expression profiles of *Acinetobacter* sp. Ver3 cells incubated under constant UV-B radiation were analyzed with respect to a dark-grown control. Roughly, the assay aims to detect proteins induced when the strain is adapted to UV-B. Exposure was set to mimic irradiation conditions during a typical day in the original environment, the Lake Verde at 4.400 m, where UV-B is supposed to reach the bacterioplankton up to a maximum intensity of 10 W m^-2^ at noon (Flores et al., 2009), thus configuring a proteome expressed in an *in vitro* UV-adapted situation.

**FIGURE 4:**
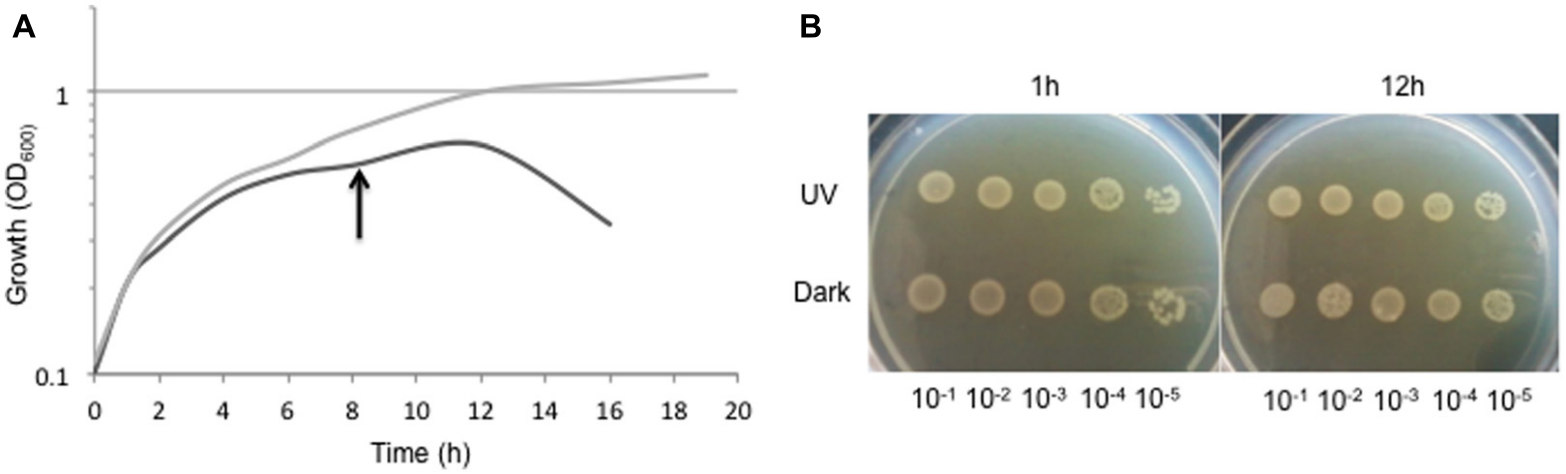
**(A)** Growth of *Acinetobacter* sp. Ver3 during 18 h of UV-exposure (black line) with respect to dark-control (gray line). Proteome was studied upon mid-exponential growth cells harvested after 8 h of incubation (arrow). **(B)** Five-μl aliquots of serial dilutions (10^-1^ to 10^-5^) of cell culture samples taken at 1 and 12 h of the growth course for both, UV-challenged cells and dark-control.

In spite of the high and constant UV dose received, Ver3 cells were able to duplicate and reached a brief stationary growth-phase and then abruptly entered a death phase (**Figure 4A**). Not-challenged cells showed a more smooth growth curve. Cell viability was maintained practically unchanged up to 12 h in both cases (**Figure 4B**). Thus, proteomic analyses were performed with mid-exponential phase cells of both treatments sampled after 8 h incubation.

The proteome of *Acinetobacter* sp. Ver3 UV-exposed cells revealed 163 spots differentially expressed but only 25 were identified by MaldiToF-MS as they resulted significantly different by a factor greater than 1.1 (Supplementary file S1). Six (spots 4, 5, 14, 23, 35, 70) and eight proteins (spots 2, 15, 33, 37, 40, 71, 76, 163) were considered up and downregulated respectively, when compared to the dark control. There were no spots exclusively appearing in the UV-challenged proteome. Closest matches to confirmed MASCOT hits in Ver3 genome sequence were obtained to improve protein identification. Interestingly, none of the detected proteins is unique to Ver3, with most of them having identities above 80% in aminoacid sequence to corresponding proteins from *A. johnsonii* DSM 6963, *A. baumannii* DSM 30007, and *A. lwoffii* DSM 2403. Likely, the enhanced resistance is related to expression or activity patterns rather than particular genes.

#### Up-Regulated Proteins

##### Proteins involved in oxidative damage response

In line with our hypothesis that oxidative damage effective response should be a likely component of Ver3 UV-resistome we found several up-regulated proteins fitting in this subsystem. Catalase (spot 35) was one of the overexpressed proteins indicating that scavenging free radicals is the first line of defense in Ver3 UV-resistome. From the genome sequence, this would be the cytoplasmic catalase (GenBank accession EZQ12194), and would be the main catalase involved in this process. Similar results were reported by Wang and Schellhorn (1995), who observed that *D. radiodurans* has high levels of catalase against effect of ultraviolet radiation and suggest that this genus possess an inducible defense mechanism against the deleterious effects of ultraviolet radiation. On the other hand, Malanga and Puntarulo (1995) describe that *Chlorella* trigger an antioxidant response related to UV-B exposure that includes an increase in the activity of enzymes SOD and catalase. Catalases were also shown to be important in polyextremophilic *Acinetobacter* strains isolated from spacecraft assembly sites, exposed to multiple stress conditions, including UV-C, H_2_O_2_, and desiccation (McCoy et al., 2012; Derecho et al., 2014). Additionally, *Acinetobacter* sp. Ver3 showed over-expression of a succinate dehydrogenase (spot 70; EZQ02211) in presence of UV radiation. This is intriguing as this protein is associated with the production of ROS (Ghosal et al., 2005) and has been shown to be downregulated as a general oxidative stress response in *Pseudomonas fluorescens* and the HepG2 cell line model systems (Mailloux et al., 2007; Lemire et al., 2010).

##### Regulator proteins

The differential proteome revealed the over-expression of a LuxR type transcriptional regulator (spot 5; EZQ11294). These proteins are key players in QS, coordinate the expression of a variety of genes, including those encoding virulence factors and antibiotics biosynthesis, motility, modulation, plasmid transfer, bioluminescence, and biofilm formation (Chen and Xie, 2011). It is gently overexpressed in the presence of UV light compared to the control, suggesting its involvement in the regulation of the expression of certain factors necessary to tolerate the UV radiation. A likely role of this regulator in biofilm formation may not be excluded in Ver3 as it forms biofilm on synthetic surfaces even under UV-challenge (Albarracín et al., unpublished). This is not the only LuxR type regulator in Ver3, a total of six proteins belong to this family (Pfam ID PF00196). Comparison with the *Acinetobacter* strains showed that these regulators have orthologs in all of them, with the exception of one (EZQ10400), which is unique in Ver3.

##### Enzymes involved in aminoacid metabolism

In the presence of UV, diaminobutyrate-2-oxoglutarate aminotransferase (DABA-AT – spot 04), dihydroxy acid dehydratase (DHAD – spot 14), and a histidyl tRNA synthetase (spot 23) were overexpressed. These proteins are implicated in amino acid metabolism at different levels. DABA-AT is involved in biosynthesis of 1,3-diaminopropane (DAP; Ikai and Yamamoto, 1997), a polyamine accumulated in members of *Acinetobacter* genus (Hamana and Matsuzaki, 1992). The protein from Ver3 (EZQ10155) presents 94% identity with the *A. baumannii* ATCC 17978 protein. DAP has been related to motility in *A. baumannii* clinical and type strains ATCC 17978 and ATCC 19606 (Skiebe et al., 2012). In *Vibrio* species it has been related to production of nor-spermidine, required for biofilm formation (Lee et al., 2009). Other polyamines are known to bind different cellular structures such as DNA, RNA, the cell wall, or the outer membrane, and to provide protection against radicals generated by oxidative stress (Wortham et al., 2007). This latter role, although not proved for DAP, might be relevant for our strain. Dihydroxy acid dehydratase is involved in biosynthesis of valine, leucine, and isoleucine. This process has been shown to be induced in *E. coli* exposed to UV-A light at mRNA level (Berney et al., 2006). The *E. coli* protein has also been shown to bear a 4Fe–4S cluster, and to be sensitive to near UV (Eisenstark, 1998). The Ver3 protein (EZQ01096) is 75% identical to DHAD from *E. coli* K-12 substr. MG1655, its overexpression might ensure providing a minimal activity required for aminoacid biosynthesis. Histidyl tRNA synthetase is responsible for the synthesis of histidyl-transfer RNA, which is essential for the incorporation of histidine into proteins. This result suggested that *Acinetobacter* increased protein synthesis (maybe specific ones with high content of histidine) required to combat the cellular stress caused by the radiation.

#### Down-Regulated Proteins

##### Enzymes involved in catabolic pathways

Upon UV-challenge, Ver3 seems to comprehensively inhibit processes related to energy production. Metabolic shutdown of tricarboxylic acid cycle and electron transport chains due to oxidative stress (Mailloux et al., 2007) might cause the down-regulation observed for a number of proteins whose role in metabolism are not clearly defined: (i) acetyl-CoA hydrolase (EZQ12320; spot 15) that catalyzes the chemical reaction: acetyl-CoA + H_2_O = CoA + acetate; (ii) the fatty acid oxidation complex sub-alpha (EZQ10967; spot 37), involved in the aerobic and anaerobic degradation of long-chain fatty acids via beta-oxidation cycle, catalyzes the formation of 3-oxoacyl-CoA from enoyl-CoA via L-3-hydroxyacyl-CoA. Beta-oxidation is the process where fatty acid molecules are broken down to generate acetyl-CoA as well as NADH, FADH_2_, which then enter the citric acid cycle and the electron transport chain, respectively; (iii) several enzymes related with the electron chain transfer: i.e., an electron transfer flavoprotein sub-beta (EZQ10505; spot 33), an electron transferring flavoprotein dehydrogenase (EZQ11819; spot 76) and pyridine nucleotide disulfide oxidoreductase (EZQ10421; spot 02). This last protein is annotated by RAST in *Acinetobacter* sp. Ver3 genome as a rubredoxin-NAD(+) reductase, which has been involved in alkane degradation in *A. bayly* ADP1 (Geissdörfer et al., 1999). Flavins are cyclic redox agents, promoters of ROS formation (Heelis, 1982), thus down-regulation of flavoproteins might reduce ROS generation. Other downregulated proteins are more directly related with ATP generation, including (iv) fructose bisphosphate aldolase class II (EZQ01582; spot 71), a protein involved in first stage of the glycolysis, that catalyzes a reversible reaction that splits the fructose 1,6-biphosphate into triose phosphates: dehydroxyacetone phosphate and glyceraldehyde 3-phosphate; and (v) adenylate kinase (EZQ02199; spot 40), is a phosphotransferase enzyme that catalyzes the interconversion of adenine nucleotides, playing an important role in cellular energy homeostasis. The activity has been reported in *A. johnsonii* 210A (Resnick and Zehnder, 2000), although its physiological role under UV stress is not clear.

##### Regulators

On spot 40, mass spectrometry indicated the presence of two different proteins. One was adenylate kinase, mentioned before, while the other was preliminary identified by MASCOT as a hypothetical protein. Nevertheless, RAST annotated the protein as “Transcriptional regulatory protein RstA,” and NCBI as “chemotaxis protein CheY” (EZQ10062). The protein does bear a CheY-like domain (Pfam PF00072), CheY proteins are single domain response regulators involved in chemotaxis (Jenal and Galperin, 2009), but in this case, the protein has additionally a DNA binding domain, suggesting a regulatory function at transcriptional level. Interestingly, downstream of this gene we found a histidine kinase sensor (EZQ10063), suggesting a two-component signal transduction system modulated by UV.

##### Enzymes involved in protein synthesis

In this case, down-regulation was seen for only the ribosome recycling factor (EZQ12436; spot 163), involved in transcriptional and translational processes. Apparently, Ver3 is able to turn on and off selective pathways related to protein synthesis; some will be much needed to counteract the effect of radiation and thus, will need upregulation as described in the previous section. As energy production is limited, some proteins with functions not useful for this particular physiological situation could be selectively not produced. In contrast (Baker-Austin et al., 2007; Belfiore et al., 2013), observed that this protein was indeed overexpressed under chemical stress.

## Conclusion

In this paper we presented the genome sequence of *Acinetobacter* sp. Ver3, a polyextremophilic gammaproteobacterium isolated from Lake Verde (4,400 m asl) together with further experimental evidence supporting the phenomenological observations regarding this bacterium ability to cope with increased UV-induced DNA damage. To the best of our knowledge, this is the first report on a genome from a polyextremophilic *Acinetobacter* strain. Comparison with genomes of other taxonomically related *Acinetobacter* strains highlighted a number of unique genes, such as a novel cryptochrome (PhlK).

An “UV-resistome” was defined, based on systems hypothetically involved in the UV phenotype of Ver3 (Di Capua et al., 2011; Albarracín et al., 2012, 2014) and other previously studied UV-resistant/tolerant strains (Malanga and Puntarulo, 1995; Eisenstark, 1998; Courcelle et al., 2001; Bosshard et al., 2010; Daly, 2012; McCoy et al., 2012). Then, genome analyses were oriented attempting to fit Ver3 genes in some of the systems as follows:

(i) UV-photoreceptor or other stress sensor and their response-regulators: the PhlK cryptochrome/photolyase (EZQ01671) might function as a photoreceptor and as regulator. The LuxR type sensor (EZQ11294), induced on growth under UV-B, might respond to UV or oxidative stress signals. OxyR, FurR, and SoxR (**Table 2**) regulators might also be indirectly related to the resistance phenotype.
(ii) UV shields: no genes were found related to special cell envelope and/or UV-absorbing pigments.
(iv) Reactive oxygen species (ROS) scavenging: a standard set of genes related to oxidative damage was found (**Table 3**). Catalase (EZQ12194) was confirmed to be upregulated upon UV-B exposure of Ver3 cells. Likely, expression patterns and enzyme efficiency would have key roles for these genes.
(v) DNA repair proteins: standard repair systems were found (**Table 2**), and they were also present in the more sensitive strains. Photorepair had been shown to be important for Ver3 (Albarracín et al., 2012). Photolyase activity would be one of the main effectors to alleviate DNA damage in light conditions, particularly the CPD dimers, which is the most abundant type of damage. PhlK might also have a role in repair.
(vi) Tolerance systems to DNA, protein, and lipid damages: Ver3 was able to sustain more pyrimidine dimer damage than other strains, suggesting the participation of some DNA repair/maintenance system (Albarracín et al., 2012). Type V error prone polymerases, encoded by several *umuDC* genes (**Table 2**), might be candidates for such function, although no mutagenic activity was confirmed yet. Other genes related to oxidative stress tolerance in membranes or proteins were also identified (**Table 3**).
(vii) Miscellaneous mechanisms. Proteins associated to amino acid and protein synthesis were observed to be up-regulated under UV-B exposure. Down-regulated proteins were related to several energy-generating pathways such as glycolysis, beta-oxidation of fatty acids and electronic respiratory chain.

**FIGURE 5:**
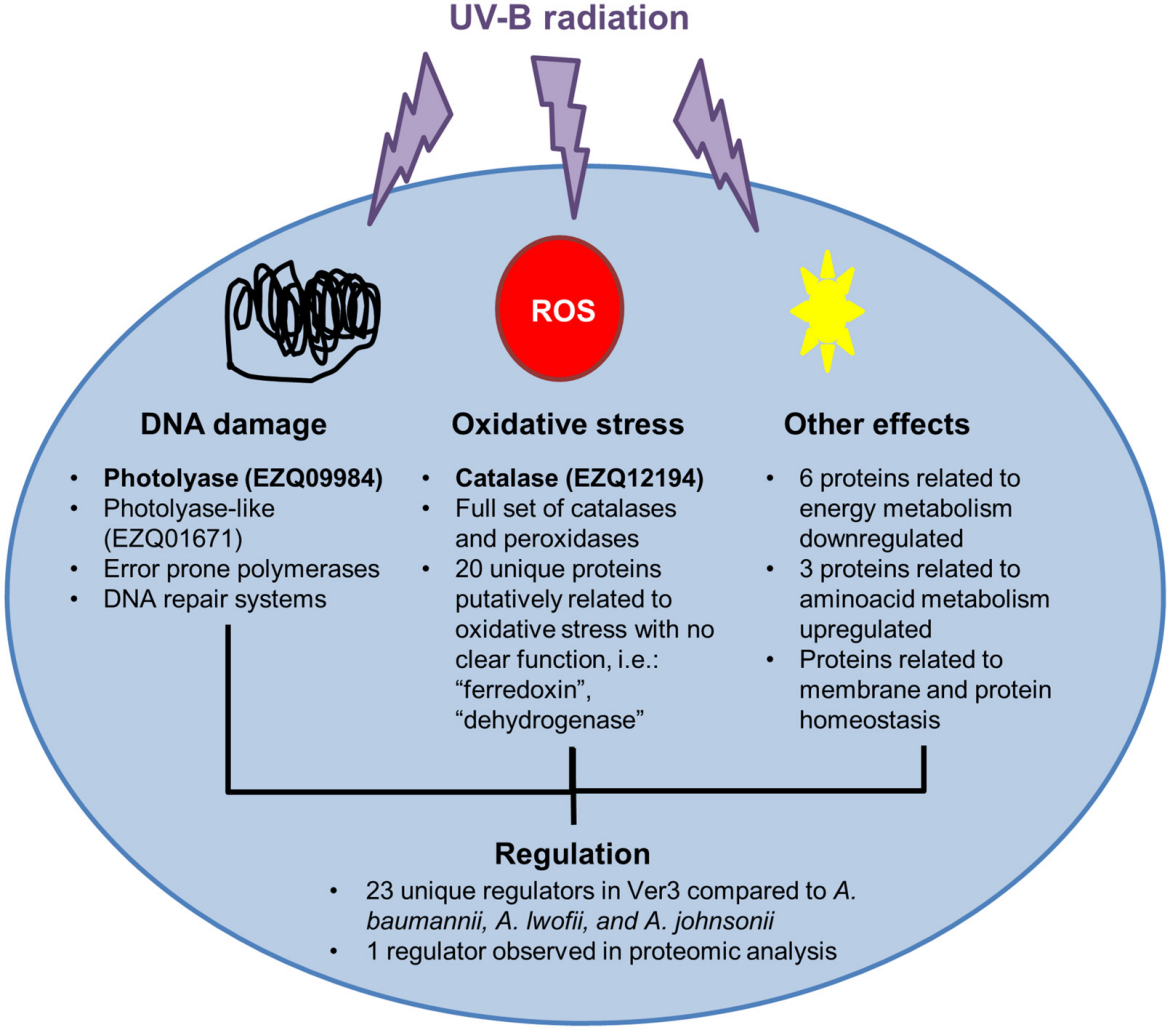
UV-resistome in *Acinetobacter* Ver3. Proteins with experimental evidence in Ver3 supporting its UV-protective function are in bold. DNA repair systems: photolyase has an important role in repairing DNA damage. Oxidative stress resistance: catalase is an inducible protein with a major role in defense against the oxidative stress. Tolerance to damage: *Acinetobacter* sp. Ver3 is able to resist damage in its DNA without losing viability. This might be due to error prone polymerases. Regulation: different systems are involved in resistance. Regulation may or may not be coordinated. Several regulators were identified; one is slightly induced while growing the strain in UV. However, none seems to directly detect UV. Regulation would respond to other signals.

Our results help to unravel the UV-resistance mechanism of *Acinetobacter* sp. Ver3 (**Figure 5**). Genomics and proteomic findings support previous work on this strain, and suggest new targets for further characterization, which might be involved in regulation and resistance to damage. More work will be required to confirm the functions of some of the genes proposed for Ver3 UV-resistome.

## Author Contributions

DK, CB, and VA designed and performed the research and wrote the paper. MG performed physiological experiments and data analysis. MF, NC, and VA obtained funding for the original project idea. All authors read and approved this manuscript.

## Footnotes

1 https://github.com/widdowquinn/scripts/blob/master/bioinformatics/calculate_ani.py

2 http://www.matrixsciende.com/search-form-select.html
