# Supplementary figures and images for: Genomic and proteomic evidences unravel the UV-resistome of the poly-extremophile Acinetobacter sp. Ver3
Source: Front Microbiol. 2015 Apr 22;6:328. doi: 10.3389/fmicb.2015.00328 (PMC4406064; doi:10.3389/fmicb.2015.00328)

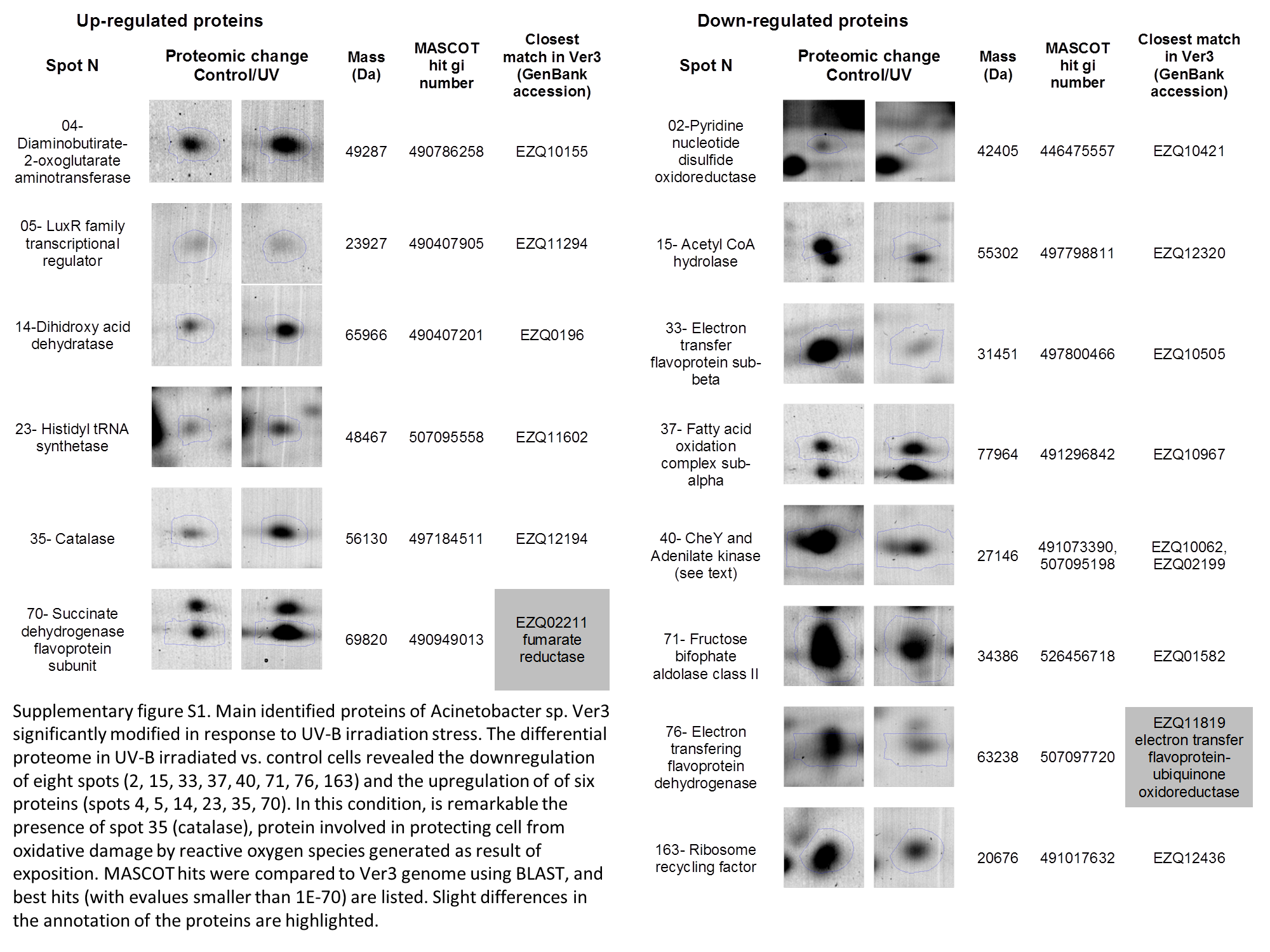

Supplement: Supplementary file 3 [file Image_1.TIF]
